# Soluble alpha-klotho and 25-hydroxivitamin D are not associated with brown adipose tissue metabolism in young healthy adults

**DOI:** 10.1007/s13105-025-01072-z

**Published:** 2025-03-11

**Authors:** Francisco J. Amaro-Gahete, Héctor Vázquez-Lorente, Guillermo Sanchez-Delgado, Jonatan R. Ruiz

**Affiliations:** 1 Department of Physiology, Faculty of Medicine, University of Granada, Granada, Spain; 2 Centro de Investigación Biomédica en Red Fisiopatología de La Obesidad y Nutrición (CIBERobn), Instituto de Salud Carlos III, 28029 Madrid, Spain; 3 Instituto de Investigación Biosanitaria, Ibs.Granada, Granada, Spain; 4 Sport and Health University Research Institute (Imuds), University of Granada, 18010 Granada, Spain; 5 Institute of Nutrition and Food Technology “José Mataix”, University of Granada, Granada, Spain; 6 Deparment of Medicine, Division of Endocrinology, Centre de Recherche du Centre Hospitalier de Sherbrooke, Université de Sherbrooke, Sherbrooke, QC Canada; 7 Pennington Biomedical Research Center, Baton Rouge, LA USA; 8 Department of Physical Education and Sports, Faculty of Sport Sciences, University of Granada, 18071 Granada, Spain

**Keywords:** Klotho protein, 25-Hydroxyvitamin D, Ageing, Brown adipose tissue

## Abstract

**Background:**

Soluble Alpha-Klotho (S-αklotho) protein and 25-Hydroxyvitamin D (25-OH-D) have emerged as potential modulators for activating and recruiting Brown Adipose Tissue (BAT). The present study aimed to investigate whether circulating S-αklotho and 25-OH-D levels are related to BAT volume, ^18^Fluorine-Fluorodeoxyglucose (^18^F-FDG) uptake, and BAT radiodensity in young healthy adults.

**Methods:**

A total of 128 participants (68% women) aged 18–25 years old participated in this cross-sectional study. Serum levels of S-αklotho were determined by a solid-phase sandwich enzyme-linked immunosorbent assay kit and 25-OH-D serum levels were analyzed using a competitive chemiluminescence immunoassay, both in blood samples collected after an overnight fast. All participants underwent a personalized cold exposure to determine their BAT volume, ^18^F-FDG uptake, and radiodensity, using a static positron emission tomography combined with computed tomography scan.

**Results:**

After adjusting for multiple covariates, serum levels of S-αklotho (all R^2^ ≤ 0.228 and P ≥ 0.364), 25-OH-D as continuous (all R^2^ ≤ 0.242 and P ≥ 0.088) or by vitamin D status (all R^2^ ≤ 0.767 and P ≥ 0.061) were not associated with either BAT volume and ^18^F-FDG uptake, or BAT radiodensity.

**Conclusion:**

Serum S-αklotho and 25-OH-D levels within the physiological range are not related to BAT-related variables in young healthy adults. Further studies are needed to fully understand the underlying mechanisms involved in BAT metabolism in humans. (ACTIBATE; ClinicalTrials.gov identifier: not applicable).

**Supplementary Information:**

The online version contains supplementary material available at 10.1007/s13105-025-01072-z.

## Introduction

The identification of novel mechanisms unravelling modifiable factors for extending health- and life-span are of clinical and scientific interest [11]. Brown adipose tissue (BAT) – a specialized tissue that maintains body temperature by oxidating glucose and lipids – is thought to be a target for decreasing the risk of obesity and for regulating energy homeostasis [8]. Feasible and effective strategies for activating and recruiting BAT at younger ages should be therefore explored, since both seem to decrease with aging [13]. In this context, the upregulation of certain hormones related to BAT metabolism may play a key role for this purpose [13].

Both α- and β-Klotho proteins have recently emerged as promising factors for human’ health with several endocrine functions [10]. α-Klotho encompasses three distinct isoforms including the intracellular, cell-membrane, and secreted/soluble (S-Klotho) forms [10]. Klotho proteins may induce browning of adipose tissue via increasing tissue specificity and metabolic action of Fibroblast Growth Factor 21 (FGF21) [10]. FGF21 is an hormonal regulator of metabolism which promotes thermogenic capacity of adipose tissues [16]. FGF21 requires Klotho protein for inducing adipose tissue browning – the downregulation of Klotho affecting metabolic regulation and thermogenic responsiveness of adipose tissues in animal models – [16]. In this line, enhancing αklotho receptor sensitivity stimulates uncoupling protein 1-independent brown fat thermogenesis through receptors outside of adipocytes in mice [4]. Currently, it is unclear whether S-αKlotho plays a role in the recruitment and activation of BAT in humans.

Vitamin D exerts its genomic and physiological effects joining to the vitamin D receptor, a member of the nuclear receptor family that regulates gene transcription [3]. Vitamin D receptor, when occupied by vitamin D, forms a heterodimer with retinoid X receptor [3]. This complex binds to the vitamin D response element in the regulatory region of the Klotho gene, leading to the transcriptional activation of this gene [3]. S-αklotho functions as a cofactor for FGF23, which suppresses parathyroid hormone (PTH) secretion and inhibits CYP27B1 activity, leading to reduced levels of circulating vitamin D and calcium [18]. S-αklotho and vitamin D are closely related in a bidirectional manner, as elevated calcium and 1,25-dihydroxyvitamin D (1,25(OH)₂D) can activate the negative feedback regulation of the αklotho–FGF23 complex, which, in turn, influences the synthesis of vitamin D [19]. Thus, vitamin D is closely related with S-αklotho protein [3]. Recent studies conducted in animal models have demonstrated that 1,25-Hydroxyvitamin D_3_ has important functions in energy metabolism processes and adipocyte biology promoting brown adipocyte differentiation at physiological concentrations through regulation of beta-oxidation and uncoupled proteins expression [23]. However, a supraphysiological increase of vitamin D concentration (via augmenting both 25-Hydroxyvitamin D (25-OH-D) and 1,25-OH-D) seems to attenuate adipose tissue browning in mice [6]. The specific impact of vitamin D, particularly the 25-OH-D form, which is the routinary measured metabolite, at physiological concentrations on human BAT metabolism remains unknown [17].

The relationship of circulating Klotho protein and vitamin D concentrations with browning BAT in humans remains unknown. Hence, the present study aimed to investigate whether circulating S-αklotho and 25-OH-D levels – both well-known modulators of the aging process – are related to BAT volume, ^18^Fluorine-Fluorodeoxyglucose (^18^F-FDG uptake), and BAT radiodensity in young healthy adults.

## Materials and methods

### Research design and participants

A total of 128 young healthy adults (n = 87 women; aged 22 ± 2 years) took part in this cross-sectional study conducted under the framework of the ACTIBATE study [22], an exercise-based randomized controlled trial (ClinicalTrials.gov identifier: not applicable). The inclusion criteria were as follows: 18 to 25 years of age, not suffering any cardiometabolic disease, presenting a body mass index (BMI) ranging from 18.5 to 35 kg/m^2^, non-smokers, not taking any medication or calcium/vitamin D supplementation, having had a stable body weight over the previous 3 months (changes < 3 kg), not being enrolled on a weight loss program or regularly exposed to cold, being physically inactive, and not having a first-degree relative history of cancer. The study protocol and informed consent procedure were performed in accordance with current ethical guidelines (Declaration of Helsinki, as revised in 2013) and approved by both the Human Research Ethics Committee of both the University of Granada (nº 924) and the Servicio Andaluz de Salud (Centro de Granada, CEI-Granada) [0838-N-2017]. All participants gave their written informed consent to be included. Participant recruitment was conducted over two consecutive years (from September 2015 to June 2016, and from September 2016 to June 2017), starting in September–December.

### Personalized cold exposure and 18F-FDG-PET/CT

The cooling protocol used and the quantification of the BAT volume and activity were performed as previously reported [1]. Briefly, subjects were sat in a cool room (19.5–20 °C) wearing a water-perfused cooling vest (Polar Products Inc., Stow, OH, USA). The water temperature was reduced ~ 2.2 °C each 10–15 min until they began shivering (determined both visually by researchers and self-reported by the participants). After 48–72 h, the participants went to the Virgen de las Nieves University Hospital, where they were again placed in a cool room (19.5–20 °C) and wore the same cooling vest but with the water temperature set ~ 4 °C above their earlier shivering threshold for 2 h. After the first hour, the subjects received an injection of ^18^F-FDG (~ 185 MBq) and the water temperature was increased by 1 °C to prevent shivering. One hour later, they were subjected to a Positron Emission Tomography/Computed Tomography (PET/CT) scan using a Siemens Biograph 16 scanner (Siemens, ER, DE). A low-dose CT scan (120 kV) was first performed for attenuation correction and anatomic localization. Immediately thereafter, one static acquisition of 2 PET bed positions (6 min each) was performed from the atlas vertebra to the mid-chest region [14]. All personalized cold exposure treatments and ^18^F-FDG-PET/CT data acquisitions were conducted according to current methodological recommendations [5].

The BAT volume, ^18^F-FDG uptake, and mean radiodensity were estimated following the BARCIST 1.0 guidelines [5] using the Beth Israel plugin (http://sourceforge.net/projects/bifijiplugins/) for the FIJI software (ImageJ, NIH, Bethesda, Maryland) [14]. This required: 1) outlining Regions Of Interest (ROIs) in the supraclavicular, laterocervical, paravertebral, and mediastinal regions from the atlas vertebra to the fourth thoracic vertebra, using a 3D-axial technique; 2) analyzing the number of pixels in the ROIs with a radiodensity range of − 190 to − 10 Hounsfield Units (HU); and 3) the determination of individualized, threshold for ^18^F-FDG standardized uptake values (SUV) [1.2/(LM (kg)/BM (kg)] [5]. BAT volume (mL) was estimated as the number of pixels within the aforementioned radiodensity range with an SUV value above the SUV threshold. Then the mean (SUVmean) and peak ^18^F-FDG activity were obatained (SUVpeak; the highest mean of the three pixels within a volume of < 1 cm^3^). The mean BAT radiodensity (HU) was calculated for all the pixels classified as BAT. Our protocol has shown a high interobserver reliability, regardless of the threshold applied to quantify BAT [5].

### Blood samples analysis

Blood samples obtained from the antecubital vein were collected in vacutainer tubes (Vacutainer SST, Becton Dickinson, PY, UK), centrifuged (i.e., 10 min at 3000 rpm) to obtain plasma (Vacutainer SST II Advance tubes, VWR International, LLC). Then, they were aliquoted, and frozen at −80ºC until analyses. The blood samples collection was performed in the morning after an overnight fast. S-αklotho serum levels (pg/mL) were determined according to a solid-phase sandwich enzyme-linked immunosorbent assay kit (Demeditec, KI, DE) strictly following the manufacturer’s recommendations. The kit uses two types of highly specific antibodies (i.e., purified mouse anti-human Klotho IgG). The optical density was measured at a wavelength of 450 – 2 nm and a standard curve was generated using known antigen concentrations. 25-OH-D serum levels (ng/mL) were analyzed using a competitive chemiluminescence immunoassay with paramagnetic particle employing a Unicel DxI 800 immunoassay system (Beckman Coulter, Brea, CA, USA). According to the Endocrine Society, vitamin D deficiency was indicated by serum 25(OH)D levels below 20 ng/mL, levels of 20–30 ng/mL were considered insufficient, while normal levels were reserved for values above 30 ng/mL [9].

### Covariates assessment

The participants’ height (m) and body mass (BM) (kg) were measured without shoes and while wearing a T-shirt and shorts using a SECA scale and stadiometer (model 799; Electronic Column Scale, HA, DE). BMI was subsequently calculated. Lean Mass (LM) (kg) and Fat Mass (FM) (expressed in kg and %), were measured using a Discovery Wi dual-energy x-ray absorptiometer (Hologic, Bedford, MA, USA). The Fat Mass Index (FMI) was determined as follows: FM (kg)/height (m)^2^, and the Lean Mass Index (LMI) as follows: LM (kg)/height (m)^2^. Sedentary time (min/day) was assessed through accelerometry using triaxial accelerometer (ActiGraph GT3X +) that participants wear on their nondominant wrist for 1 week [1]. Alkaline phosphatase (U/L), creatinine (mg/dL), and uric acid (mg/dL) were obtained from routinary hospital analytics.

### Statistical analysis

The Kolmogorov–Smirnov test, visual check of histograms, Q-Q, and box plots were used to verify and accept the normal distribution of all variables. The descriptive parameters were reported as mean and standard deviation. Unpaired-samples T-Student test was performed to study differences between BAT variables (i.e., BAT volume, BAT SUV_mean_, BAT SUV_peak_, and BAT mean radiodensity) by sex. Multiple linear regression models were conducted to assess the relationship between I) S-αklotho and 25-OH-D levels, II) S-αklotho and 25-OH-D levels with BAT-related variables, and III) serum 25-OH-D levels by vitamin D status categories with BAT-associated parameters. A basic model was adjusted for sex (men or women), age (years), and the date when the PET-CT scan was performed (year/month/day). A fully-adjusted model was additionally adjusted for LMI (in kg/m^2^), FMI (in kg/m^2^), sedentary time (in min/day), alkaline phosphatase (in U/L), creatinine (in mg/dL), and uric acid (in mg/dL). The analyses were performed using the Statistical Package for Social Sciences (SPSS, v. 22.0, IBM SPSS Statistics, IBM Corporation). For the graphical plots, GraphPad Prism 8 software (GraphPad Software, San Diego, CA, USA) was used. A *p*-value < 0.05 was considered statistically significant.

## Results

Table 1 describes the baseline characteristics of the study’ participants together and by sex. Significant differences by sex were observed for BM, BMI, LMI, FMI, sedentary time, S-αklotho, and 25-OH-D (all P < 0.05). All BAT parameters were similar in both sexes (all P > 0.05). S-αklotho and 25-OH-D showed no relationship after adjusting for multiple covariates (R^2^ = 0.175; P = 0.739, Fig. S1).

**Table 1:** Characteristics of the study subjects

	**All** **(n = 128)**		**Men** **(n = 41)**		**Women** **(n = 87)**	
**Age (years)**	22.0	(2.10)	22.4	(2.20)	21.8	(2.10)
**Anthropometrics and body composition**						
Body mass (kg)	71.2	(17.2)	85.3	(18.2)	64.5	(12.0)***
Body mass index (kg/m^2^)	24.9	(4.8)	27.5	(5.5)	23.8	(3.9)***
Lean mass index (kg/m^2^)	14.6	(2.5)	17.3	(2.1)	13.4	(1.4)***
Fat mass index (kg/m^2^)	8.9	(3.1)	8.62	(3.6)	9.04	(2.7)
Fat mass (%)	35.9	(7.3)	31.0	(7.6)	38.2	(5.9)***
**Sedentary time (min/day)**	791.2	(2.1)	807.7	(2.2)	783.4	(52.9)*
**Alkaline phosphatase (U/L)**	72.7	(19.4)	80.2	(20.0)	69.1	(17.5)
**Creatinine (mg/dL)**	4.7	(1.2)	5.7	(1.1)	0.7	(0.1)
**Uric acid (mg/dL)**	0.8	(0.1)	0.9	(0.1)	4.2	(0.9)
**S-αklotho serum levels (pg/mL)**	839.7	(559.3)	642.4	(438.8)	932.7	(587.5)**
**25-OH-D serum levels (ng/mL)**	26.4	(8.2)	23.7	(6.84)	27.8	(8.5)**
**Brown adipose tissue**						
BAT volume (mL)	70.9	(59.2)	84.0	(69.9)	64.8	(52.7)
BAT SUV_mean_	3.8	(1.9)	3.40	(1.5)	4.03	(2.1)
BAT SUV_peak_	11.5	(8.3)	10.5	(7.8)	12.0	(8.6)
BAT mean radiodensity (HU)	−60.2	(9.7)	−59.9	(9.9)	−60.3	(9.7)

***Notes:*** Data are shown as means (standard deviation). Abbreviations: BAT; Brown Adipose Tissue, HU; Hounsfield Units, S-αklotho; Soluble Alpha-Klotho, SUV; Standardized Uptake Value, 25-OH-D; 25-Hydroxyvitamin D. Significant differences between sex were obtained by independent-sample T-test. *P ≤ 0.05, **P ≤ 0.01, ***P ≤ 0.001

### Soluble alpha klotho protein is not associated with BAT-related variables

**Fig. 1:**
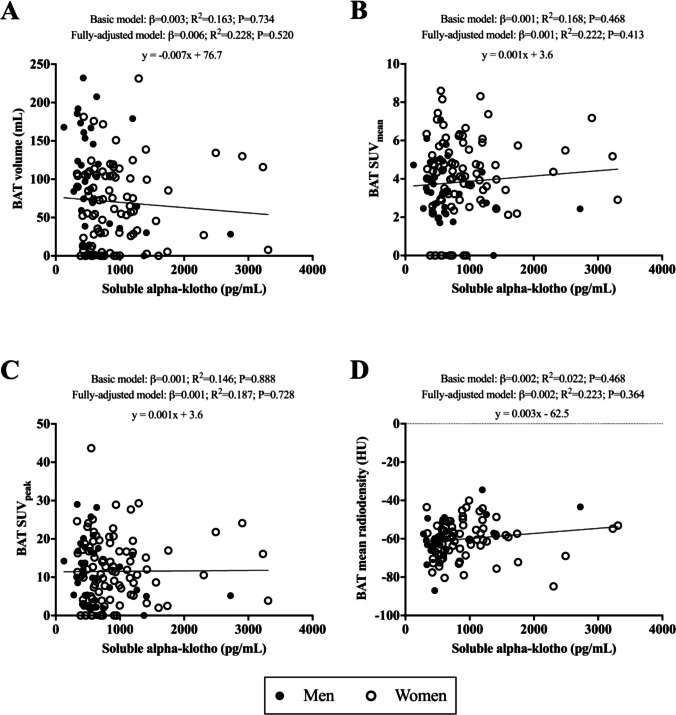
Association of soluble alpha-klotho serum levels with BAT volume (Panel **A**; n = 128), BAT SUV_mean_ (Panel **B**; n = 128), BAT SUV_peak_ (Panel **C**; n = 128), and BAT mean radiodensity (Panel **D**; n = 98), in young adults. β standardized regression coefficient; R^2^ and P are provided for multiple linear regression analyses. Basic model was adjusted for sex (men or women), age (years), and the date when the PET-CT scan was performed (year/month/day). The fully-adjusted model was additionally adjusted for lean mass index (in kg/m^2^), fat mass index (in kg/m^2^), sedentary time (min/day), alkaline phosphatase (in U/L), creatinine (in mg/dL), and uric acid (in mg/dL). Significance was set at *p*-value < 0.05. Abbreviations: BAT; Brown Adipose Tissue, SUV; Standardized Uptake Value, HU; Hounsfield units

We observed no significant associations of S-αklotho serum levels with BAT volume, BAT SUV_mean_, BAT SUV_peak_, and BAT mean radiodensity (all R^2^ ≤ 0.228 and P ≥ 0.364, Fig. 1, Panels A-D) in the fully-adjusted model.

### No relationship between 25-hydroxyvitamin D and BAT-associated parameters

**Fig. 2:**
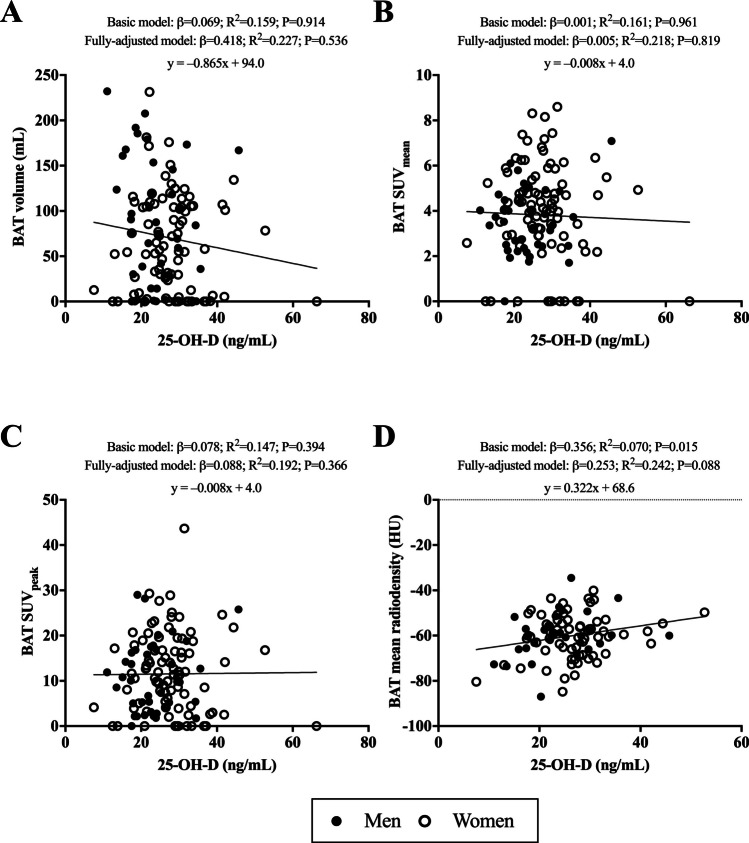
Association of 25-OH-D serum levels with BAT volume (Panel **A**; n = 128), BAT SUV_mean_ (Panel **B**; n = 128), BAT SUV_peak_ (Panel **C**; n = 128), and BAT mean radiodensity (Panel D; n = 98), in young adults. β standardized regression coefficient; R^2^ and P are provided for multiple linear regression analyses. Basic model was adjusted for sex (men or women), age (years), and the date when the PET-CT scan was performed (year/month/day). The fully-adjusted model was additionally adjusted for lean mass index (in kg/m^2^), fat mass index (in kg/m^2^), sedentary time (in min/day), alkaline phosphatase (in U/L), creatinine (in mg/dL), and uric acid (in mg/dL). Significance was set at *p*-value < 0.05. Abbreviations: BAT; Brown Adipose Tissue, HU; Hounsfield units, SUV; Standardized Uptake Value, 25-OH-D; 25-Hydroxyvitamin D

After adjusting for multiple covariates, 25-OH-D serum levels showed no significant relationship with BAT volume, BAT SUV_mean_, BAT SUV_peak_, and BAT mean radiodensity (all R^2^ ≤ 0.242 and P ≥ 0.088, Fig. 2, Panels A-D). Moreover, 25-OH-D serum levels by categories of vitamin D status deficiency, insufficiency, and sufficiency, showed no significant relationship with BAT volume, BAT SUV_mean_, BAT SUV_peak_, and BAT mean radiodensity (all R^2^ ≤ 0.767 and P ≥ 0.061, Table S1).

## Discussion

The present work sought to elucidate, for the first time, the potential association of serum S-αklotho and 25-OH-D levels with BAT-related parameters (i.e., BAT volume, ^18^F-FDG, and radiodensity) assessed strictly following the most updated methodological recommendations for BAT assessment in humans. After adjusting for multiple covariates, serum S-αklotho and 25-OH-D concentrations showed no relationship with BAT-related variables following cold exposure in young healthy adults. Taken together, these findings suggest an unclear role of S-αklotho and 25-OH-D as potential modulators of BAT metabolism, at least in young healthy adults.

### Soluble alpha-klotho protein is not associated with BAT-related variables

Research examining the relationship between Klotho protein and BAT in humans are currently limited, and the available information primarily stems from β-Klotho in animal and cellular models. A recent study investigating the effects of β-Klotho protein gene ablation in mice led to a decrease in energy expenditure, without concomitant disruption of energy intake, resulting in a positive energy balance increasing the likelihood of developing obesity due to reduction of FGF21 [16]. In this regard, Klotho knockout mice have been observed to present less energy expenditure, body temperature and gene expression of BAT (i.e., UCP-1 and PEPCK genes) compared to wild-type mice [15]. Moreover, reductions of β-Klotho concentrations in cellular lines have been related with brown adipocyte dysfunction and premature death as a result of increased inflammatory damage upon BAT-specific proteins (i.e., uncoupling and Klotho proteins) [20]. Regretfully, the previous-mentioned studies were performed in animal or cellular models, and only considered β-Klotho protein concentrations, facts that could explain the lack of relationships between S-αklotho protein and BAT-related parameters in our study’ cohort. Moreover, there could be different physiological effects in extreme conditions (i.e., total absence of S-αklotho protein) compared with those observed in homeostatic ranges [15].

### No relationship between 25-hydroxyvitamin D and BAT-associated variables

There is limited research exploring the relationship between circulating 25-OH-D and BAT-related outcomes in humans, with the majority of existing studies being conducted in animal models. Despite vitamin D is involved in improving muscle mass and decreasing adipose tissue content [7], we observed no relationship between serum 25-OH-D levels and BAT-related variables. These findings contrasts with those obtained in a previous study that used a transgenic mice model with hypermetabolism and 25-OH-D deficiency, where the repletion of 25-OH-D_3_ levels ameliorated adipose tissue browning [7]. Moreover, serum 25-OH-D_3_ repletion in mice suffering kidney damage (i.e., characterized by basal low serum concentrations of 25-OH-D) additionally had the same previous-mentioned response upon ameliorating adipose tissue browning [21]. The potential reasons why our results do not fully concur with the above-mentioned findings could be explained because most of these previous studies were conducted in animal models suffering different pathological conditions. Further well-designed interventional studies are needed to elucidate the role of vitamin D on BAT metabolism in healthy humans.

### Limitations and strengths

Our results should be considered with caution since some limitations arise. This is a cross-sectional study, and no causal relationships can be established. Despite being the most used technique to assess BAT, a single static ^18^F-FDG-PET/CT scan has several limitations that might not allow for the accurate estimation of BAT volume and function [2]. The sample was composed of young healthy adults aged 18–25 from Spain and therefore the results should be limited to this age group and country. Hence, interventional studies in aged populations or in other countries are needed to confirm our findings. Of note, outdoor activity, which may increase blood 25-OH-D concentrations and potentially influence BAT activity, was not assessed in this study. Moreover, additional biomarkers such as FGF21, FGF23, UCP1, and 1,25-OH-D, which could have offered a more comprehensive understanding of the underlying mechanisms described in this study, were not evaluated due to limitations in biological sample availability. It should be mentioned that an important strength of this study was the use of cutting-edge methods for BAT assessment following the most updated recommendations [12]. Moreover, this work is pioneering at studying this interesting topic in humans shedding light on the role of S-αKlotho and 25-OH-D as potential factors related to BAT metabolism in young adults.

## Conclusion

Our findings indicate that serum S-αklotho and 25-OH-D levels within the physiological range are not related to BAT variables in young healthy adults. Further intervention studies are needed to fully comprehend the underlying mechanisms involved in BAT activation and recruitment and their relationship with health and lifespan in humans.

## Supplementary Information

Below is the link to the electronic supplementary material.

Supplementary file1 (DOCX 1105 KB)

Supplementary file2 (DOCX 108 KB)

## Data Availability

No datasets were generated or analysed during the current study.
