# Supplementary material for: Soluble alpha-klotho and 25-hydroxivitamin D are not associated with brown adipose tissue metabolism in young healthy adults
Source: J Physiol Biochem. 2025 Mar 11;81(2):291–8. doi: 10.1007/s13105-025-01072-z (PMC12279559; doi:10.1007/s13105-025-01072-z)
Supplement: Supplementary file 1 — Supplementary file1 (DOCX 1105 KB) [file 13105_2025_1072_MOESM1_ESM.docx]

**
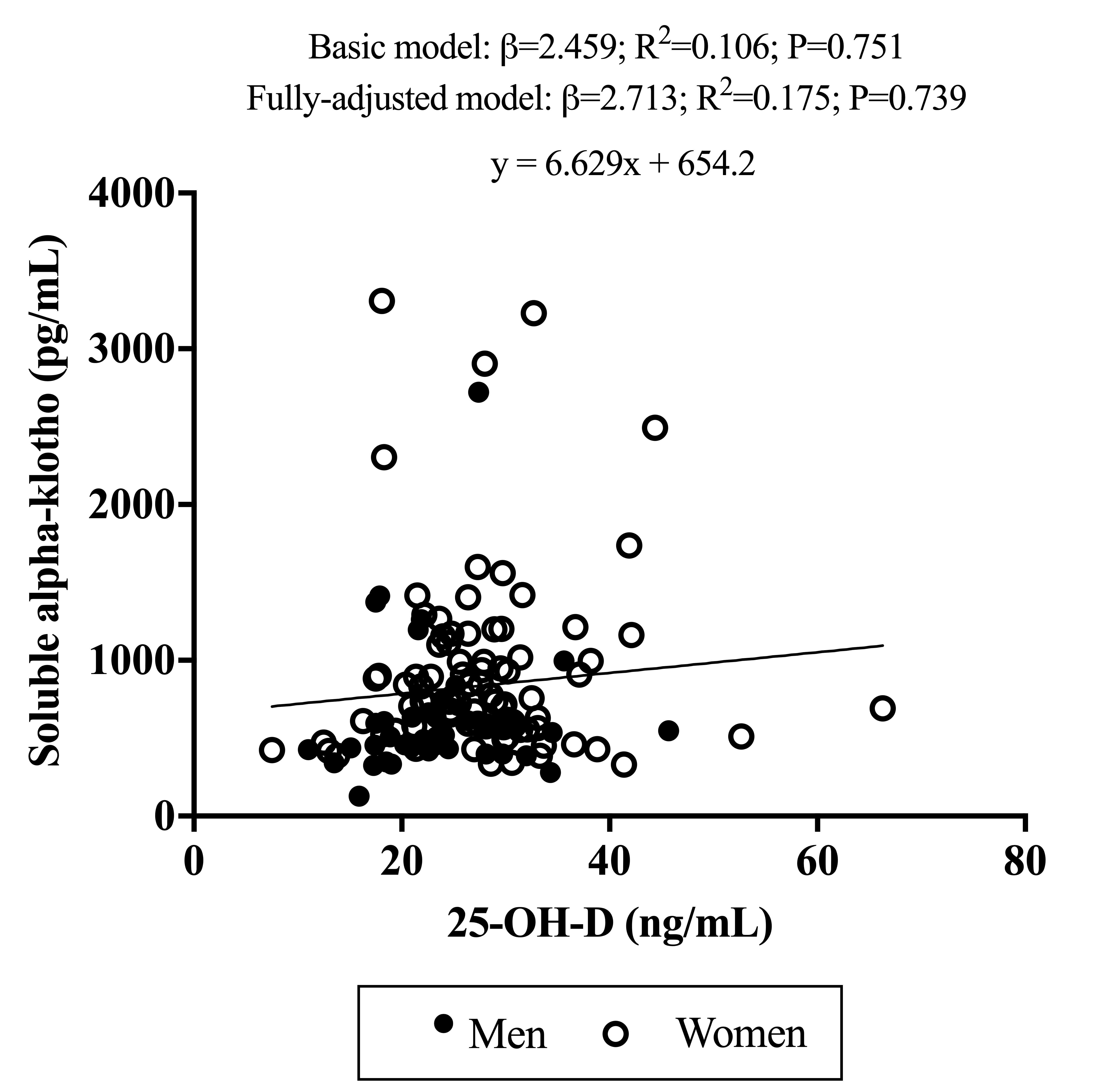
**

**Fig. S1.-** Association between 25-OH-D and soluble-alpha klotho serum levels. β standardized regression coefficient; R^2^ and P are provided for multiple linear regression analyses. Basic model was adjusted for sex (men or women), age (years), and the date when the PET-CT scan was performed (year/month/day). The fully-adjusted model was additionally adjusted for lean mass index (in kg/m^2^), fat mass index (in kg/m^2^), sedentary time (min/day), alkaline phosphatase (in U/L), creatinine (in mg/dL), and uric acid (in mg/dL). Significance was set at *p*-value <0.05. Abbreviations: BAT; Brown Adipose Tissue, HU; Hounsfield units, SUV; Standardized Uptake Value, 25-OH-D; 25-Hydroxyvitamin D.
