# Supplementary material for: Soluble alpha-klotho and 25-hydroxivitamin D are not associated with brown adipose tissue metabolism in young healthy adults
Source: J Physiol Biochem. 2025 Mar 11;81(2):291–8. doi: 10.1007/s13105-025-01072-z (PMC12279559; doi:10.1007/s13105-025-01072-z)
Supplement: Supplementary file 2 — Supplementary file2 (DOCX 108 KB) [file 13105_2025_1072_MOESM2_ESM.docx]

| **Table S1.-** Association of 25-OH-D serum levels by vitamin D status with BAT volume BAT SUV_mean,_ BAT SUV_peak_, and BAT mean radiodensity | | | | | | | | | |
| --- | --- | --- | --- | --- | --- | --- | --- | --- | --- |
| **Characteristic** | **Vitamin D status** | | | | | | | | |
|  | **Deficiency (n=24)** | | | **Insuficiency (n=69)** | | | **Suficiency (n=35)** | | |
|  | **β** | **R^2^** | ***p*-value** | **β** | **R^2^** | ***p*-value** | **β** | **R^2^** | ***p*-value** |
| **BAT volume (mL)** |  |  |  |  |  |  |  |  |  |
| Basic model | 1.084 | 0.373 | 0.831 | –2.772 | 0.237 | 0.217 | 1.337 | 0.366 | 0.395 |
| Fully-adjusted model | 3.135 | 0.575 | 0.613 | –2.610 | 0.286 | 0.266 | 2.551 | 0.544 | 0.185 |
| **BAT SUV_mean_** |  |  |  |  |  |  |  |  |  |
| Basic model | 0.172 | 0.377 | 0.164 | –0.065 | 0.204 | 0.373 | 0.053 | 0.332 | 0.344 |
| Fully-adjusted model | 0.142 | 0.511 | 0.377 | –0.060 | 0.360 | 0.394 | 0.071 | 0.561 | 0.276 |
| **BAT SUV_peak_** |  |  |  |  |  |  |  |  |  |
| Basic model | 0.687 | 0.305 | 0.221 | –0.209 | 0.195 | 0.499 | 0.278 | 0.272 | 0.384 |
| Fully-adjusted model | 0.772 | 0.420 | 0.309 | –0.210 | 0.271 | 0.509 | 0.316 | 0.480 | 0.410 |
| **BAT mean radiodensity (HU)** |  |  |  |  |  |  |  |  |  |
| Basic model | 1.361 | 0.452 | 0.086 | –0.086 | 0.030 | 0.869 | 0.066 | 0.018 | 0.848 |
| Fully-adjusted model | 2.332 | 0.767 | 0.061 | –0.214 | 0.255 | 0.672 | –0.025 | 0.120 | 0.962 |
| β standardized regression coefficient; R^2^ and P are provided for multiple linear regression analyses. Basic model was adjusted for sex (men or women), age (years), and the date when the PET-CT scan was performed (year/month/day). The fully-adjusted model was additionally adjusted for lean mass index (in kg/m^2^), fat mass index (in kg/m^2^), sedentary time (min/day), alkaline phosphatase (in U/L), creatinine (in mg/dL), and uric acid (in mg/dL). Significance was set at *p*-value <0.05. Abbreviations: BAT; Brown Adipose Tissue, HU; Hounsfield units, SUV; Standardized Uptake Value, 25-OH-D; 25-Hydroxyvitamin D. | | | | | | | | | |
